# Sulforaphane enhances progerin clearance in Hutchinson–Gilford progeria fibroblasts

**DOI:** 10.1111/acel.12300

**Published:** 2014-12-16

**Authors:** Diana Gabriel, Daniela Roedl, Leslie B Gordon, Karima Djabali

**Affiliations:** 1 Department of Medicine, Epigenetics of skin Aging and Institute for Medical Engineering, Technische Universität München (TUM)Garching bei München, Germany; 2 Department of Pediatrics, Alpert Medical School of Brown University and Hasbro Children's HospitalProvidence, RI, USA; 3 Boston Children's Hospital and Harvard UniversityBoston, MA, USA

**Keywords:** lamins, progeria, progerin, proteostasis, senescence, sulforaphane

## Abstract

Hutchinson–Gilford progeria syndrome (HGPS, OMIM 176670) is a rare multisystem childhood premature aging disorder linked to mutations in the *LMNA* gene. The most common HGPS mutation is found at position G608G within exon 11 of the *LMNA* gene. This mutation results in the deletion of 50 amino acids at the carboxyl-terminal tail of prelamin A, and the truncated protein is called *progerin*. Progerin only undergoes a subset of the normal post-translational modifications and remains permanently farnesylated. Several attempts to rescue the normal cellular phenotype with farnesyltransferase inhibitors (FTIs) and other compounds have resulted in partial cellular recovery. Using proteomics, we report here that progerin induces changes in the composition of the HGPS nuclear proteome, including alterations to several components of the protein degradation pathways. Consequently, proteasome activity and autophagy are impaired in HGPS cells. To restore protein clearance in HGPS cells, we treated HGPS cultures with sulforaphane (SFN), an antioxidant derived from cruciferous vegetables. We determined that SFN stimulates proteasome activity and autophagy in normal and HGPS fibroblast cultures. Specifically, SFN enhances progerin clearance by autophagy and reverses the phenotypic changes that are the hallmarks of HGPS. Therefore, SFN is a promising therapeutic avenue for children with HGPS.

## Introduction

Hutchinson–Gilford progeria syndrome (HGPS, OMIM 176670) is a rare segmental premature aging disorder associated with rapid growth deceleration in childhood (Gordon *et al*., 2014). The most frequent mutation G608G (GGC>GGT) occurs within exon 11 of LMNA encoding a mutant lamin A protein called progerin, which is missing 50 amino acid near the carboxyl terminus of prelamin A (Eriksson *et al*., 2003). This truncation removes the ZMPSTE24 cleavage site from the lamin A processing pathway (Sinensky *et al*., 1994; Young *et al*., 2006), resulting in a persistent progerin farnesylation. Progerin consequently is tightly anchored to the nuclear envelope, disrupting the nuclear lamina and causing nuclear blebbing, heterochromatin disorganization, and accumulation of DNA double-strand breaks (DSB) in HGPS patient cells, as well as in cells in which the progerin is expressed by transgenic methods (Goldman *et al*., 2004; Liu *et al*., 2006; Manju *et al*., 2006; Gordon *et al*., 2014).

Several studies have shown that cells or mouse models of progeria after treatment with either farnesyltransferase inhibitor (FTI), statins, or isoprenylcysteine carboxyl methyltransferase (ICMT) inhibitor could reverse some of the phenotypic changes occurring in these cells or tissues (Fong *et al*., 2006; Capell *et al*., 2008; Yang *et al*., 2008; Ibrahim *et al*., 2013). To date, three clinical trials involving children with HGPS have been initiated using the drugs mentioned above to block prelamin A maturation (Gordon *et al*., 2014). The identification of new compounds that can boost progerin degradation will significantly improve recovery for children affected by this syndrome.

To date, there are very few compounds that are known to enhance the protein degradation systems. One of them is sulforaphane (SFN, 1-isothiocyanato-4-(methylsulfinyl)-butane), a potent inducer of antioxidant enzymes, and is found in cruciferous vegetables, especially in young broccoli sprouts (Verkerk *et al*., 2009). SFN reduces oxidative stress through the activation of antioxidant response pathways (Verkerk *et al*., 2009) and promotes proteostasis (Kwak *et al*., 2007; Gan *et al*., 2010). This study tested the ability of SFN to induce progerin clearance and improve disease phenotype in HGPS fibroblast cultures.

## Results

### Proteomics analysis of HGPS nuclei

**Figure 1:**
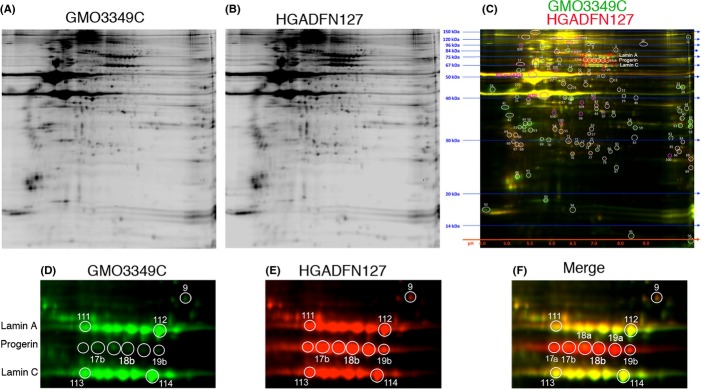
Two-dimensional difference in gel electrophoresis (2D-DIGE) analysis of the nuclear proteome in Hutchinson–Gilford progeria syndrome (HGPS) fibroblasts. HGPS and normal fibroblast nuclei were labeled separately with CyDye DIGE fluors. Normal nuclear extract with Cy2 (A) and HGPS nuclear extract with Cy3 (B). Equal protein amounts of control and HGPS proteins were simultaneously separated on a single 2D gel. Two independent experiments, with two controls and two HGPS cell lines, were performed. Representative gel images of control (GMO3349C) in green and HGPS (HGADFN127) in red are shown (C). Images D, E, and F correspond to enlarged gel images of the separated signals at the positions of A-type lamin protein spots, as indicated.

We quantitatively compared nuclear protein extracts from HGPS fibroblasts and normal dermal fibroblasts (passages 12–14) using two-dimensional difference in gel electrophoresis (2D-DIGE) followed by mass spectrometry (MS) of the selected protein spots (Fig.1). Two independent analyses using different HGPS and control cell lines were performed (see Procedures). The representative 2D-DIGE protein profiles of HGADFN127 versus control GMO3349C are shown (Fig.1). On the merged image, equally expressed proteins are visualized in yellow, proteins with a higher expression in HGPS nuclei in red, and proteins highly expression in control in green (Fig.1C).

An average of 1000 protein spots were detected on gels, and 114 spots were found to increase or decrease by more than 1.5-fold in HGADFN127 nuclei (Fig.1). The most significantly increased or decreased proteins were picked and identified using matrix-assisted laser desorptionhionization time-of-flight/time-of flight (MALDI-TOF/TOF) MS as described in Experimental procedures. Tandem MS fragmentation spectra were acquired for each of the ten most abundant ions present in each protein spot and submitted for a search to identify proteins from the NCBInr database. Candidates with either a protein score confidence interval (CI) % or Ion C.I. % >95 were considered significant. Among the most altered proteins identified were spots 17–19 a, and b (Fig.1C,F), which represent progerin protein. Progerin was 10- to 20-fold increased in HGPS; however, progerin was also detected in normal nuclei as a weaker green spot at position 17b and a stronger spot at position 18a (Fig.1D). Collectively, 2D-DIGE analyses indicate that progerin protein spots are biomarkers of HGPS and that trace amounts of progerin are detectable in normal nuclei.

### Proteins differentially represented in the HGPS nucleome

From two independent 2D-DIGE analyses, we identified 75 protein spots by MS that were differentially represented and found 28 proteins included in both studies (Table1). Functional analysis of these 28 proteins using bioinformatics software (Ingenuity Systems, Redwood City, CA, USA) revealed functional links to at least four major biological categories: (i) amino acid metabolism and post-translational modification, (ii) collagen function, (iii) signal transduction and protein processing, and (iv) protein degradation and chaperone proteins. This list of protein contained lamin A and C, progerin, and lamin B1 (Table1). Additionally, we found several molecular chaperones (Hsp27, Hsp70, and Hsp90) that interact with misfolded proteins, and other proteins including PSMC2 (the 26S protease regulatory subunit 7), BAG2 (Bcl2-associated athanogene), and valosin-containing protein (VCP), which are components of the proteolytic pathways (Table1).

**Table 1:** Proteins identified from the two-dimensional difference in gel electrophoresis (2D-DIGE) by mass spectrometry (MS)

Proteins	Swiss Prot Nr	Fold change	Subcellular localization	Function
26S protease regulatory subunit 7 (PSMC2)	P35998	−1.44	Nucleus/Cytoplasm	Subunit of the 26S proteasome complex, involved in degradation of proteins, hydrolysis of ATP, and proliferation of cells
Annexin A1	P04083	2.88	Nucleus/Cytoplasm	Calcium/phospholipid binding protein, responsible for: cellular development, cellular growth and proliferation, fusion of vesicles, actin cytoskeleton
ATP-dependent RNA helicase (DDX1)	Q92499	2.01	Nucleus/Cytoplasm	Is a putative RNA helicase, involved in cellular development, growth and proliferation, has exonuclease and helicase activity
BAG family molecular chaperone regulator 2 (Bag2)	O95816	2.42	Nucleus/Cytoplasm	Co-Chaperone, involved in folding of proteins, metabolism of proteins, Inhibition of Ubiquitin ligase CHIP
Chaperone protein HSP90 beta (Hsp90b)	P08238	−1.57	Nucleus/Cytoplasm	Chaperone protein, involved in protein folding and protein degradation
Chromosome 14 open reading frame 166	Q549M8	1.75	Nucleus	Is a protein-coding gene, involved in binding to RNA polymerase II regulation of transcription
Collagen, type VI, alpha 1	P12109	−2.31	Endoplasmic reticulum	Collagen metabolic process and response to amino acid stimuli
Cysteine and glycine-rich protein 1	P21291	3.56	Nucleus	Encodes LIM-domain proteins, responsible for: cellular development, cellular growth and proliferation
EH-domain containing 3	Q9NZN3	−1.59	Nucleus/Cytoplasm	Role in endocytic transport and GTP catabolic process
Enolase 1, (alpha)	P06733	−1.44	Nucleus/Cytoplasm	Glycolytic enzyme, features: DNA binding, magnesium ion binding, phosphopyruvate hydratase activity, protein binding, has transcription corepressor activity
Eukaryotic translation elongation factor 2	Q9GZV4	−1.56	Nucleus/Cytoplasm	Elongation factor, mRNA binding, protein biosynthesis and transport.
FHL1	Q13642	−3.79	Nucleus/Cytoplasm	Four and a half LIM domain protein Role in cellular development, growth, proliferation and differentiation.
Guanine nucleotide-binding protein subunit beta-2-like 1 (GNB2L1)	P63244	−2.64	Nucleus/Cytoplasm	Receptor for activated protein kinase C (PKC), involved in cellular development, growth and proliferation, positive regulation of proteasomal ubiquitin-dependent protein catabolic process
Heat-shock protein beta-1 (Hsp27)	P04792	−1.89	Nucleus/Cytoplasm	Chaperone, responsible for folding of proteins, degradation of proteins, antiapoptosis, initiation of translation of mRNA and ubiquitin binding
Heat-shock-related 70 kDa protein 2 (Hsp70)	P54652	2.68	Nucleus/Cytoplasm	Chaperone, responsible for unfolded protein binding, refolding of proteins, dissociation and reassociation of 26S proteasome
Lamin A	P02545	–	Nucleus	Lamin A component of the nuclear lamina
Lamin C	P02545	–	Nucleus	Lamin C component of the nuclear lamina
Lamin A Variant, Progerin	P02545	10–20	Nucleus	Mutant lamin A linked to Hutchinson–Gilford progeria syndrome
Lamin B1	P20700	−1.99	Nucleus	Lamin B component of the nuclear lamina
Leprecan-like 2	Q8IVL6	1.88	Nucleus/Endoplasmic reticulum	Prolyl 3-hydroxylase 3, metal ion binding, oxidoreductase activity. Located in Endoplasmic reticulum
MAPK8, Mitogen-activated protein kinase 8 interacting protein 1	P45983	2.01	Nucleus/Cytoplasm	Serine/threonine-protein kinase involved in various processes such as cell proliferation, differentiation, migration, transformation and programmed cell death
Prolyl 4-hydroxylase subunit alpha-1	P13674	−1.55	Endoplasmic reticulum	Key enzyme in collagen synthesis, involved in cellular development, growth and proliferation
Protein disulfide isomerase P4HB	P07237	−1.13	Endoplasmic reticulum	Catalyze the formation, breakage of disulfide bonds, and reductase activity. Function as chaperone and isomerase
Pyruvate kinase, muscle (PKM)	P14618	−2.21	Nucleus/Cytoplasm	Glycolytic enzyme that catalyzes the transfer of a phosphoryl group from phosphoenolpyruvate (PEP) to ADP, generating ATP. Stimulates POU5F1-mediated transcriptional activation
Ras and Rab interactor 2 (RIN2)	Q8WYP3	−1.13	Cytoplasm/Nucleus	GTPase activator activity
Serpin H1	P50454	−2.89	Endoplasmic reticulum	Binds specifically to collagen. Could be involved as a chaperone in the biosynthetic pathway of collagen.
Transketolase (TKTL1)	P51854	−3.5	Nucleus/Cytoplasm	Catalyzes the transfer of a two-carbon ketol group from a ketose donor to an aldose acceptor, via a covalent intermediate with the cofactor thiamine pyrophosphate.
Valosin-containing protein (VCP)	P55072	−2.02	Nucleus/Endoplasmic reticulum/Cytoplasm	VCP binds ubiquitinated proteins and is necessary for the export of misfolded proteins. Role in DNA damage response via RNF factors and maybe recruited to stalled replication forks by SPRTN.

A total of 75 protein spots of interest were selected on two independent 2D-DIGE analyses and identified based on peptide mass fingerprint mapping (using MS spectra) and peptide fragmentation mapping (using MS/MS spectra). The identified proteins were analyzed using Ingenuity Pathways Analysis software (Ingenuity Systems), and their functional associations are indicated.

### Proteasome activity is altered in HGPS

**Figure 2:**
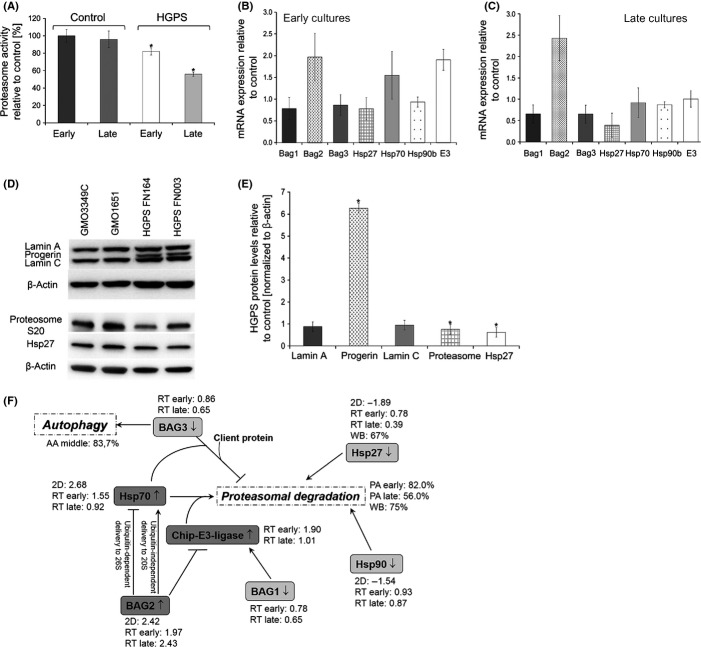
Protein degradation activities are reduced in Hutchinson–Gilford progeria syndrome (HGPS) cells. (A) Proteasome activity was defined by measuring the chymotrypsin-like proteasome activity in four controls and four HGPS cell lines using Suc-LLVY-AMC as a substrate. The percentage of activity was calculated relative to early passage control cultures. Data are expressed as the mean ± SD. *Indicates values that are significantly different from controls (*P <* *0.05; *n* = 4). (B and C) Encoding mRNA levels of the indicated proteins were determined in total mRNA preparations isolated from early (<15) and late (>18) passages control and HGPS cells by real-time PCR. All values are presented as the mean ± SD (*P <* *0.05; *n* = 4). (D) Representative Western blots of lamin A/C, progerin, proteasome subunit S20 C2, Hsp27 and β-actin in control and HGPS total cell extracts. (E) Quantifications of lamin A, lamin C, progerin, proteasome S20 C2, and Hsp27 levels normalized to β-actin are presented as the fold change relative to control cells (*n* = 4; *P <* *0.05). (F) A schematic representation of protein alterations linked to protein degradation pathways in HGPS cells. The proteins identified by two-dimensional difference in gel electrophoresis (2D-DIGE) (BAG1, BAG2, BAG3, Chip-E3-ligase, Hsp70, Hsp90, and Hsp27) are shown. Their levels were determined by 2D-DIGE followed by mass spectra analysis (2D), real-time PCR (RT) and Western blot analyses (WB), as indicated. Proteasome activity (PA) and autophagy (AA) in HGPS cells relative to control cells are indicated. All values represent the values of HGPS cells compared with control cells.

To further validate our proteomics data regarding protein degradation pathways, we investigated proteasome activity in four HGPS and four normal fibroblast lines in early and late passages (Fig.2A). Although the normal cells exhibited no significant changes in proteasome activity, on average, the HGPS cells demonstrated a decrease in activity that worsened in late passages (Fig.2A). Gene expression profiling of components linked to degradation systems indicated that the HGPS cells exhibited an approximately twofold increase in BAG2 [late cultures (*P *=* *0.073)] and decreased levels in BAG1 (0.65-fold; *P *=* *0.063) and BAG3 (0.65-fold; *P *=* *0.066) (Fig.2B,C). Moreover, the mRNA levels of the chaperones Hsp27 were decreased in late HGPS cultures (0.39-fold, *P *=* *0.080), whereas the levels of Hsp90 and Hsp70 remained similar to control cells (Fig.2B,C). However, E3 mRNA levels were found increased by 1.90-fold (*P *=* *0.082) at early passage in HGPS cells relative to control cells (Fig.2B), while at late passages in HGPS cells, E3 levels decreased to the levels of control cells (Fig.2B). In summary, the real-time PCR data were consistent with the proteomics data.

Next, Western blot analyses of total protein extracts from HGPS and normal cells were performed (Fig.2D,E). While the levels of lamin A and C remained constant, progerin was highly expressed in HGPS cells (Fig.2D,E). The protein levels of Hsp27 (0.62-fold, *P *=* *0.008) and the 20S proteasome subunit C2 (20SC2) (0.75-fold, *P *=* *0.003) were significantly reduced in HGPS cells (Fig.2D,E).

The proteins associated with the degradation systems, including the chaperones and the BAG proteins (Table1), were integrated into a schematic representation according to their functional relationships, as indicated in Fig.2F. In particular, BAG1 is a regulator of the proteasomes, whereas BAG3 is a regulator of macroautophagy (Gamerdinger *et al*., 2009), and BAG2 is a specific inhibitor of the chaperone-associated ubiquitin ligase CHIP E3 ligase as indicated (Fig.2B,C) (Gamerdinger *et al*., 2009).

### Sulforaphane enhances protein degradation in HGPS cells

Sulforaphane (1-isothiocyanato-4-methylsulfinylbutane) is a plant-derived isothiocyanate (ITC) that was previously reported to enhance proteasome activity by inducing expression of the small heat-shock protein Hsp27 and the 26S proteasome subunit PSMB5 (Kwak *et al*., 2007; Gan *et al*., 2010). We therefore tested whether SFN treatment might also exert beneficial effects on cellular homeostasis in HGPS.

**Figure 3:**
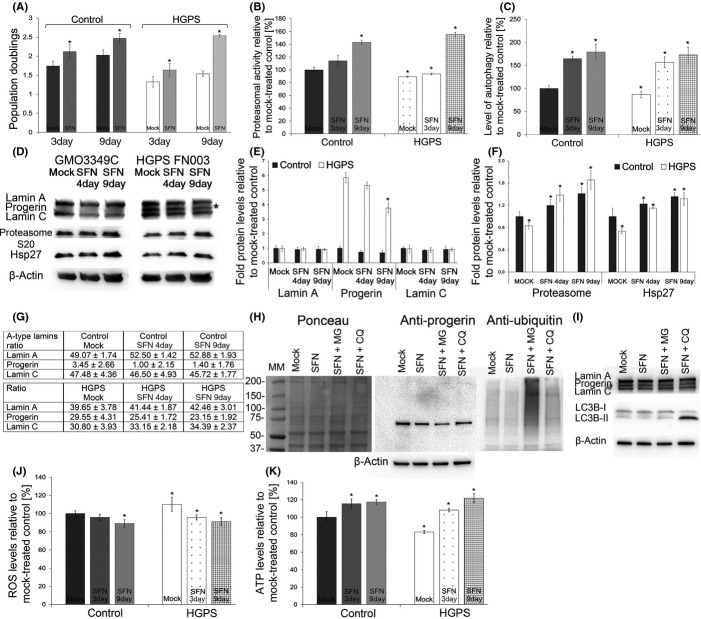
Sulforaphane (SFN) treatment rescues the Hutchinson–Gilford progeria syndrome (HGPS) cellular phenotypes. (A) Population doubling levels were calculated as described in the Materials and Methods for control and HGPS cells that were mock treated (vehicle DMSO) or treated daily with 1 μmSFN for a period of 3 or 9 days. (B) Proteasome activity was determined by measuring chymotrypsin-like proteasome activity in four control and four HGPS fibroblast lines using Suc-LLVY-AMC as a substrate. The percentage of activity was calculated relative to the activity in mock-treated control cells. Data are expressed as the mean ± SD (*P <* *0.05; *n* = 4). (C) The same cells and culture conditions as in (B) were used to determine autophagy activity by measuring monodansylcadaverine (MDC) levels using fluorescence photometry, as indicated in the Procedures. Data are presented as the mean ± SD (*P <* *0.05; *n* = 4). (D) Representative Western blots for lamin A/C, proteasome subunit 20S C2, Hsp27, and β-actin in total cell extracts isolated from mock-treated control and HGPS cells and cells treated with 1 μmSFN daily for a period of 4 or 9 days. (E and F) Quantification of Westerns (D) for lamin A, lamin C, progerin, proteasome subunit 20S C2 and Hsp27 levels normalized to β-actin and presented as the fold change relative to the levels in mock-treated control cells (*P <* *0.05; *n* = 5). (G) The proportions of lamin A, progerin, and lamin C were determined within each sample analyzed by Western blotting with anti-lamin A/C antibody in panel (D). (H) Representative Western blot of HGPS cell lysates from cultures that were mock treated or treated with SFN or SFN plus MG132 (MG) or with chloroquine (CQ). Left panel corresponds to Ponceau red staining of the blot probed sequentially with antibodies specific for progerin, ubiquitin, and β-actin (*n* = 3). (I) Representative Western blot of the same culture conditions as in (H) probed with antibodies specific for lamin A/C, LC3B-I and LC3B-II and β-actin (*n* = 3). (J) Intracellular reactive oxygen species (ROS) levels were determined by measuring oxidized dichlorofluorescein (DCF) levels using a 2′,7′-dichlorofluorescein diacetate (DCFDA) cellular ROS detection assay, as described in the Procedures. Data represent the mean ± SD (*P <* *0.05; *n* = 3) compared with mock-treated counterparts. (K) Cellular ATP levels were measured using a CellTiter-Glo luminescence ATP assay, as described in the Procedures. Data represent the mean ± SD (*P <* *0.05; *n* = 3) relative to mock-treated counterparts.

We first assessed the toxicity of SFN by applying different concentrations to control fibroblast cultures (Fig. S1). Because treatment with 5 μm SFN led to >1% cell death among control cells after 48 h, we chose to use 1 μm SFN, which resulted in reduced cell death relative to mock-treated control cells. In all subsequent experiments, the cells were fed daily with fresh medium supplemented with 1 μm SFN or vehicle (DMSO). The HGPS and control cells treated with 1 μm SFN exhibited a significantly increased growth rate on day 3 compared with their mock-treated counterparts (Fig.3A), and the proliferation defect in the HGPS cells was ameliorated after 9 days of SFN treatment (Fig.3A).

Next, we tested the effect of SFN on proteasome activity (Fig.3B). Control cells treated with SFN demonstrated proteasome activity that increased with time relative to mock-treated controls. Although HGPS cells initially exhibited decreased proteasome activity compared with normal cells, SFN treatment also significantly increased the cells' proteasome activity after 9 days of treatment.

Cells rely on two proteolytic systems to maintain proteostasis: the lysosomal system (or autophagy) and the ubiquitin–proteasome system (Gamerdinger *et al*., 2009). Therefore, we also investigated the levels of autophagy in HGPS cells. In particular, early-passage HGPS cells exhibited a significant (16.4%, *P *=* *0.0010) decrease in autophagy compared with control cells (Fig.3C), and SFN treatment significantly enhanced autophagy by day 3 in both control (1.64-fold, *P *=* *0.012) and HGPS (1.79-fold, *P *=* *0.050) cells, (Fig.3C). We then determined whether SFN could enhance the clearance of progerin in HGPS cells. Western blot analyses showed that the levels of lamin A and C levels remained relatively constant in both control and HGPS cells treated with SFN; conversely, progerin signals in SFN-treated HGPS cells were decreased by 13% (*P *=* *0.343) by day 4 and by nearly 40% (*P *=* *0.007) by day 9 compared with signals in mock-treated control cells (Fig.3D,E). To further assess the impact of SFN on A-type lamins, the proportions of lamin A, lamin C and progerin were determined within each sample (Fig.3G), with the proportion of progerin being significantly decreased by day 9 (21.7%; *P *=* *0.036) in SFN-treated HGPS cells relative to mock-treated HGPS cells (Fig.3G). Collectively, these data indicated that SFN ameliorates the status of A-type lamins in HGPS cells.

**Figure 4:**
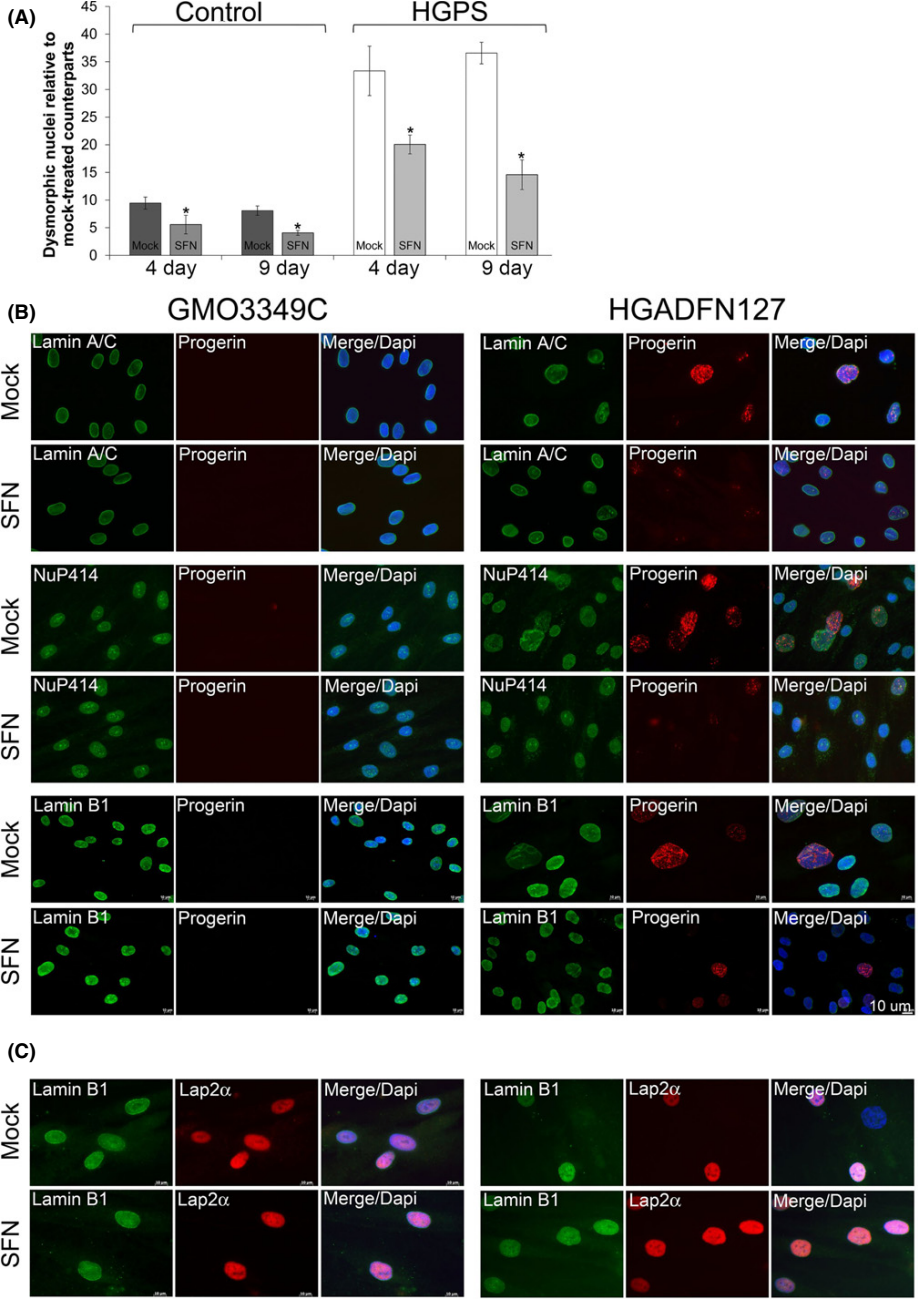
The effect of sulforaphane (SFN) on the distribution of progerin and nuclear proteins in normal and HGPS fibroblasts. (A) Frequency of misshapen nuclei (dysmorphic) in three control and three HGPS fibroblast lines after 4 or 9 days of treatment with either vehicle or SFN (1 μm). The bars indicate the mean frequency of misshapen nuclei. An average of 800 nuclei were examined for each control and HGPS cell line, and treatment, and each experiment was repeated three times. (B) Immunocytochemistry using antibodies directed against the indicated proteins (progerin, lamin A/C, a nuclear pore proteins (Nup414), and lamin B1) was performed on normal (GMO3349C) and HGPS (HGADFN127) cells mock-treated or SFN-treated cells for a period of 9 days. (C) The same cells and conditions as in (A) were immunolabeled with anti-LAP2α and anti-lamin B1 antibodies. Scale bar: 10 μm.

To further understand the mechanisms by which SFN enhances proteostasis, we investigated the levels of the 20S C2 subunit. Four days of SFN treatment induced a significant increase in the levels of the 20S C2 subunit in both control (1.17-fold, *P *=* *0.023) and HGPS cells (1.65-fold, *P *=* *0.018) (Fig.3F), and Hsp27 levels were also increased after SFN treatment in these cells (control 1.21-fold, *P *=* *0.038; HGPS 1.66-fold, *P *=* *0.025) (Fig.4F). Collectively, our data indicate that SFN induces upregulation of the protein components involved in protein degradation pathways in control and HGPS cells.

To determine which protein degradation pathway is responsible for the clearance of progerin in SFN-treated HGPS cells, cells were treated with 1 μm SFN for 5 days and then exposed to SFN in combination with 1 μm MG132, a proteasome inhibitor, or 25 μm chloroquine diphosphate, an inhibitor of autophagy, for 12 h (Fig.3H). The HGPS cells exhibited an increased amount of ubiquitinated proteins in response to MG132 (Fig.3H), and the administration of chloroquine resulted in a significant increase in LC3B-II (Fig.3I), a specific marker of autophagosomes that accumulates when autophagy is inhibited (Gamerdinger *et al*., 2009). In contrast, LC3B-II levels were barely detectable in HGPS cells treated with SFN alone or with MG132, indicating that autophagy remained active (Fig.3I). Additionally, progerin levels were reduced in SFN-treated cells by 28% (*P *=* *0.032) and furthermore reduced by 37% (*P *=* *0.027) in SFN-MG132-treated cells by comparison to mock-treated counterparts (Fig.3H). This result is consistent with previous study indicating that autophagy is stimulated by proteasome inhibition (Milani *et al*., 2009). The progerin levels in cells exposed to SFN and chloroquine for 12 h remained similar to those in SFN-treated cells (Fig.3H), that is, this duration of SFN plus chloroquine treatment was not long enough to induce a detectable increase in progerin levels. Collectively, these data indicate that SFN stimulates autophagy and thereby enhances progerin clearance in HGPS cells.

Hutchinson–Gilford progeria syndrome proteomics analysis indicated a significant decrease in the levels of FHL-1 (four and a half LIM protein 1) (Table1). As mutations in the FHL-1 gene have been previously linked to another laminopathy, Emery-Dreifuss muscular dystrophy (EDMD) (Gueneau *et al*., 2009), we investigated FHL-1 protein levels in total fibroblast extracts (Fig. S2). Of the three isoforms (FHL-1A, FHL-1B, and FHL-1C) encoded by the FHL-1 gene, FHL-1C was the most significantly decreased in HGPS fibroblasts (Fig. S2). SFN treatment was efficient in upregulating FHL-1 levels in HGPS cells, as detected by Western blotting and immunocytochemistry (Fig. S2).

Previous studies have demonstrated increased levels of reactive oxygen species (ROS) and decreased levels of ATP in HGPS cells (Viteri *et al*., 2010). As SFN is known to have antioxidant properties, we next tested its effect on ROS and ATP levels in HGPS fibroblasts. We found that ROS levels were increased in early-passage HGPS cultures and were reduced in the presence of SFN (Fig.3J). In contrast, ATP levels were significantly decreased in HGPS cells compared with normal cells (Fig.3K), and SFN treatment induced an increase in intracellular ATP levels in both control and HGPS cells. These data indicate that the antioxidant activity of SFN ameliorates reduced energy levels in HGPS cells.

### SFN treatment improves nuclear morphology in HGPS fibroblasts

Well-defined abnormalities in HGPS cells include nuclear envelope alterations and reduced levels of the nuclear components, lamin B1, and LAP2α (Goldman *et al*., 2004; Shimi *et al*., 2011). We first investigated the impact of SFN on the nuclear shape, including the nuclear blebs and invaginations observed in dysmorphic HGPS nuclei (Goldman *et al*., 2004). SFN treatment reduced the frequency of nuclear blebbing in both control and HGPS cells after 4 days, with further reductions after 9 days of treatment (Fig.4A). Next, immunohistochemistry with an anti-progerin antibody revealed progerin accumulation in the most dysmorphic HGPS nuclei and localization to the nuclear envelope, with aggregated staining in certain areas (Fig.4B). In contrast, progerin staining in normal-shaped nuclei exhibited weak signals, with very few dots or foci, and other nuclei exhibited no signal. In SFN-treated HGPS cultures, both the number of brightly labeled nuclei and the signal intensity in progerin-positive nuclei were reduced compared with those in mock-treated HGPS cultures. Additionally, the percentage of nuclei with bright progerin staining was reduced from an average of 38% in mock-treated HGPS cultures to 15% in SFN-treated HGPS cultures. However, the distribution of the nuclear pores was not altered by SFN treatment (Fig.4B). Lamin B1 signals in the most dysmorphic HGPS nuclei were very low to barely detectable (Fig.4C), whereas bright progerin-positive HGPS nuclei were associated with weak lamin B1 staining. After SFN treatment, the number of HGPS nuclei exhibiting reduced lamin B1 signals was reduced concomitantly with a decreased number of bright progerin-positive nuclei (Fig.4C). Finally, a low lamin B1 signal always coincided with a low LAP2α signal (Fig.4B). Thus, the frequency of nuclei with low levels of lamin B1 and LAP2α staining in SFN-treated HGPS cells was reduced. Together, these findings indicated that SFN normalized the levels of lamin B1 and LAP2α by reducing progerin levels in HGPS nuclei.

### Treatment of HGPS fibroblasts with SFN reduces the levels of DNA damage

**Figure 5:**
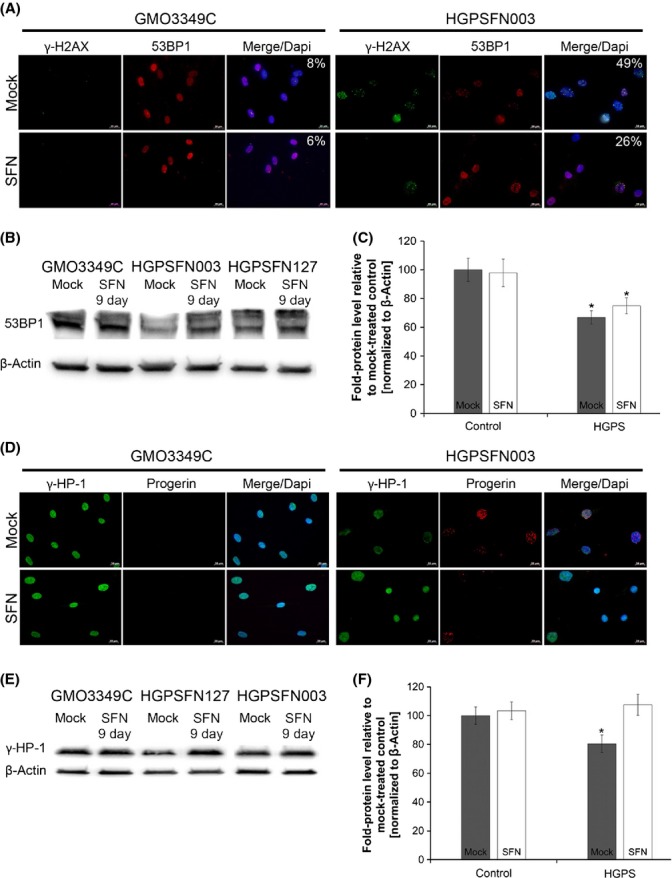
Sulforaphane (SFN) ameliorates the levels of DNA damage in Hutchinson–Gilford progeria syndrome (HGPS) cells. (A) Immunocytochemistry using antibodies directed against indicated proteins (γ-H2AX and 53BP1) was performed on normal (GMO3349C) and HGPS (HGADFN003) cells mock-treated or SFN-treated cells for 9 days. The percentage of nuclei showing γ-H2AX foci is indicated (*n* = 4). Scale bar: 20 μm. (B) Western blot evaluation of 53BP1 levels in control and HGPS cells that were treated as in (A). (C) Quantification of 53BP1 levels normalized to β-actin and presented as the fold change relative to control cells (*P <* *0.05; *n* = 3). (D) Immunocytochemistry using antibodies directed against indicated proteins (HP1-γ and progerin) was performed on normal (GMO3349C) and HGPS (HGADFN003) cells mock-treated or SFN-treated for 9 days. (E) Western blot evaluation of HP1-γ levels in control and HGPS cells that were treated as in (D). (F) Quantification of HP1-γ levels normalized to β-actin and presented as the fold change relative to control cells (*P <* *0.05; *n* = 3).

HGPS cells accumulate endogenous DNA damage, and particularly DSBs, with passage in culture (Liu *et al*., 2005; Scaffidi & Misteli, 2005). Thus, we determined the basal levels of DNA damage in mock-treated fibroblasts after staining with antibodies against the phosphorylated form of γH2A.X, which detects DSBs. The number of HGPS nuclei harbouring γH2A.X foci was 49% on average, which was significantly higher than the number in normal fibroblasts (8%) (Fig.5A). However, 9 days of SFN treatment significantly reduced the number of nuclei with γH2A.X foci, to an average of 26% (Fig.5A). These results indicate more efficient DNA damage repair in HGPS cells in the presence of SFN. Moreover, consistent with previous studies showing that the levels of the DNA repair factors 53BP1 and RAD51 are altered in HGPS cells (Liu *et al*., 2005, 2008), Western blot, and immunostaining analyses indicated that 53BP1 (Fig.5A–C) and Rad51 (Fig. S3) levels were decreased in HGPS cells. However, in the presence of SFN, the levels of these factors were increased, and their nuclear distribution was normalized (Fig.5 and Fig. S3). Collectively, these findings indicate that SFN treatment improves DNA damage repair in HGPS cells.

Hutchinson–Gilford progeria syndrome cells exhibit a loss of heterochromatin and reduced levels of chromatin proteins, such as heterochromatin protein 1 (HP1) (Goldman *et al*., 2004; Scaffidi & Misteli, 2006). In accordance with the cited studies, we found that HP1γ levels were decreased in HGPS cells compared with normal fibroblasts (Fig.5D). However, in the presence of SFN, the levels of HP1γ were restored in HGPS cells, as observed by immunohistochemistry studies (Fig.5D) and Western blot analysis (Fig.5E,F).

### Sulforaphane in combination with a FTI exhibits no synergistic effect

Several studies have shown that FTI treatment can ameliorate the nuclear shape abnormalities observed in HGPS cells (Capell *et al*., 2005; Glynn & Glover, 2005; Toth *et al*., 2005; Yang *et al*., 2005; Marji *et al*., 2010). Therefore, we investigated whether SFN in combination with lonafarnib (an FTI) could exert a synergistic effect on HGPS cells (Fig. S4). Control and HGPS cells were treated either separately with SFN (1 μm) or the FTI at a previously described concentration (1.5 μm) (Marji *et al*., 2010) or with a combination of both (Fig. S4). FTI treatment alone induced the accumulation of the prelamin A protein in both normal and HGPS cells (Fig. S4A). Progerin levels were significantly reduced only in SFN-treated HGPS cells (Fig. S4), whereas FTI treatment in combination with SFN did not lead to a reduction in progerin levels (Fig. S4). Immunohistochemical analyses also indicated that HGPS cells exhibited a more ovoid nuclear shape in the presence of FTI or FTI plus SFN (Fig. S4). However, a significant number of cells harbored donut-shaped nuclei after 4 days of FTI treatment (Fig. S4C, arrows), as reported previously (Verstraeten *et al*., 2011; Wang *et al*., 2012). The average frequency of donut-shaped nuclei was 7% on day 4 of FTI treatment and had increased to approximately 35% by day 9. After 9 days of FTI or FTI plus SFN treatment, the frequencies of donut-shaped nuclei were similar, and 75% of these nuclei were binucleated (Fig. S4D). Moreover, FTI with or without SFN decreased the growth rate of both control and HGPS cells (Fig. S4E). Collectively, these observations indicate that further drug titration studies will be needed to determine the potential use of an FTI in combination with SFN.

### Long-term SFN treatment further ameliorates the HGPS cellular phenotype

**Figure 6:**
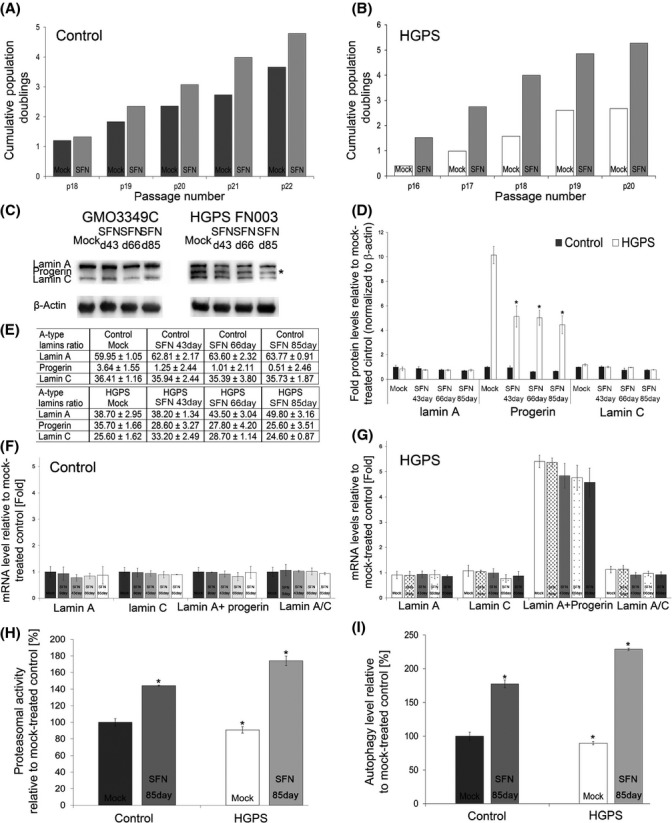
Effect of sulforaphane (SFN) on long-term control and Hutchinson–Gilford progeria syndrome (HGPS) fibroblast cultures. (A) Long-term cultures of control (GMO3349C and GMO1651C) and (B) HGPS (HGADFN127 and HGADFN003) cells that were mock treated or treated with SFN (1 μm). The cumulative population doubling was calculated at each indicated passage as described in the Procedures. (C) Western blot evaluation of A-type lamin (lamin A, lamin C, and progerin) levels in control and HGPS cells that were mock treated or treated with SFN every 2 days for the period indicated (a representative image is shown: *n* = 3). Blots were probed with antibodies against lamin A/C and β-actin. (D) Densitometric analysis of lamin A, lamin C, and progerin signals. Data represent the mean ± SD with respect to mock-treated control cells after the values were normalized to the β-actin signal (*n* = 3). (E) The proportions of lamin A, progerin, and lamin C were determined within each sample analyzed by Western blotting with anti-lamin A/C antibody as shown in (C). (F and G) The mRNA levels of the indicated proteins in total mRNA preparations isolated from normal (F) and HGPS (G) cells were determined using real-time PCR. Cells were either mock treated or treated with 1 μm SFN every 2 days for the period indicated. All values are presented as the mean ± SD (*P* < 0.05; *n* = 3). (H) Proteasome activity was defined by measuring chymotrypsin-like proteasome activity in the same cells as in (A) using Suc-LLVY-AMC as a substrate. Cells were either mock treated or treated with 1 μmSFN, every 2 days for 85 days as indicated. The percentage of activity was calculated relative to mock-treated control. Data are expressed as the mean ± SD (*P <* *0.05; *n* = 3). (I) The same cells and culture conditions as in (H) were used to determine autophagy activity by measuring monodansylcadaverine (MDC) levels by fluorescence photometry, as described in the Methods. Data are presented as the mean ± SD (*P <* *0.05; *n* = 3).

To test the long-term effect of SFN on HGPS and normal fibroblasts, cultures were passaged several times and treated every other day with 1 μm SFN or vehicle for several months (Fig.6). The proliferation rates of normal and HGPS cells demonstrated sustained increases in growth in the presence of SFN (Fig.6A,B). Western blot analyses showed a significant and sustained decrease in progerin levels in SFN-treated HGPS cells in comparison with normal fibroblasts during the 85-day period of treatment (Fig.6C,D). The status of A-type lamins, as indicated by the proportions of lamin A, progerin, and lamin C within each sample, also showed that progerin levels were significantly decreased in the presence of SFN (Fig.6E). Importantly, the proportions of lamin A increased with these decreased levels of progerin in the treated HGPS cells (Fig.6E), thus indicating that SFN ameliorated the ratio of A-type lamins in SFN-treated HGPS cells. Gene expression profiling of A-type lamins mRNA levels indicated that the overall levels remained constant under all conditions (Fig.6F,G). As proteasome activity and autophagy levels were increased in both control and HGPS cells treated with SFN for 85 days (Fig.6H,I), our results indicated that SFN treatment induced a sustained increase in proteostasis in the cells during long-term treatment. Consequently, progerin clearance in SFN-treated HGPS cells remained high during the entire 12-week period of treatment.

## Discussion

The 2D-DIGE analysis of isolated nuclei from control and HGPS cells showed significant changes in the HGPS nucleome. The abundance of progerin in HGPS nucleome makes this lamin A variant the biomarker of HGPS cells. Moreover, very low levels of progerin were also detected in normal nuclei. Prior studies showed that cells derived from unaffected individuals sporadically use the same cryptic splice site in lamin A, and several immunohistochemistry studies using anti-progerin antibodies have shown that progerin is present in cells from elderly individuals (McClintock *et al*., 2007; Olive *et al*., 2010). Therefore, it is possible that normal cells might accumulate progerin over time and might manifest similar cellular alterations to those observed in HGPS cells.

Hutchinson–Gilford progeria syndrome nucleome exhibits changes in components involved in the proteolytic systems, suggesting that HGPS cells lose proteostasis. These changes include the heat-shock proteins (Hsp70, Hsp90, and Hsp27) that are linked to protein degradation via the proteasomal or lysosomal pathways. These activities decline with aging and contribute to the development of several age-related pathologies such as Alzheimer's disease, Parkinson's disease, cataracts, and vascular disease (Koga *et al*., 2011). HGPS cells appear to share a similar mechanistic link as they also show a decline in protein degradation activities.

Proteasomes are present in both the nucleus and cytoplasm (Breusing & Grune, 2008). Similarly to a previous study, we showed a decrease in the 26S subunit C2 and reduced proteasome activity (Viteri *et al*., 2010). We also found changes in the expression of several members of the BAG family in HGPS cells. These cochaperones are implicated in protein quality control and regulate the proteasome and autophagy pathways (Gamerdinger *et al*., 2009). As observed previously, we report that autophagy levels are decreased in HGPS cells and appear to exacerbate the cellular phenotype in late cultures (Cao *et al*., 2011a). Strategies aimed at reducing progerin levels in HGPS cells using molecular approaches or drug treatments indicate a significant amelioration of the phenotype in progeria cells (Gordon *et al*., 2014). Previously, AIMP3, an aminoacyl-tRNA synthetase-interacting multifunctional protein, was shown to interact with lamin A and promote its degradation by proteasomes (Oh *et al*., 2010), while progerin and lamin C, which are lacking AIMP3 interaction site undergo degradation by autophagy (Oh *et al*., 2010). Herein, we provide further evidence for progerin degradation by autophagy in HGPS cells treated with SFN.

SFN belongs to the ITCs, which are derived from cruciferous vegetables, such as broccoli. SFN induces several cellular protective mechanisms, including induction of the Keap1-Nrf2 pathway (Baird & Dinkova-Kostova, 2011). Moreover, SFN has been shown to enhance proteasome activity by upregulating several proteasomal subunits and Hsp proteins (Gan *et al*., 2010; Vallanat *et al*., 2010). Consistently, we report that SFN treatment of HGPS cells increased the expression of components of the proteasome system and of several Hsps and cochaperones, thereby increasing proteasome activity and autophagy. The reduction of progerin levels by SFN in HGPS cells led to nuclear envelope normalization, increased proliferation, and proteostasis, all of which were sustained and even improved over time in the presence of SFN.

Three previous studies have shown that rapamycin, an inhibitor of the mammalian target of rapamycin (mTOR) pathway, significantly reduces progerin levels via autophagy in HGPS cells (Cao *et al*., 2011b; Cenni *et al*., 2011; Blondel *et al*., 2014), whereas a fourth study detected no reduction (Ibrahim *et al*., 2013). Collectively, these studies support the idea that therapies aimed at increasing autophagy could ameliorate the HGPS cellular phenotype.

Because HGPS cells accumulate DNA DSBs, progerin has been thought to affect the rate of DSB repair (Liu *et al*., 2005). Although restoring certain phenotypic effects of the disease, treatment of HGPS fibroblasts with FTIs does not reduce DNA damage (Liu *et al*., 2006). However, one study has shown that the ROS scavenger *N*-acetyl cysteine (NAC) can reduce the basal levels of DSBs (Richards *et al*., 2011). In the present study, we provide evidence indicating that SFN treatment can also reduce the levels of DNA damage in HGPS cells.

Because several preclinical studies and clinical trials examining FTI use in HGPS have shown many ameliorations (Gordon *et al*., 2014), we tested the potential use of an FTI in combination with SFN to treat HGPS cells. At the concentrations used by others in this study, we found that this cotreatment reduced cell growth. However, further titration of both drugs will be needed to determine their potential synergistic effect.

In conclusion, our results not only confirm the link between the level of progerin in HGPS nuclei and the severity of the associated phenotypic alterations but also demonstrate that enhanced proteostasis can be achieved in HGPS cells. To our knowledge, this is the first report showing that SFN, a small bio-compound, can improve proteasome activity and autophagy, reduce the levels of DNA damage, and improve the growth rate of HGPS cells. SFN might also be beneficial for normal cells. Our findings support the use of SFN, with its broad range of cytoprotective activities, as a valuable therapeutic strategy for children with HGPS and possibly other age-related conditions.

## Experimental procedures

### Cell culture and drug treatments

Fibroblasts from patients with HGPS were obtained from The Progeria Research Foundation Cell and tissue Bank (http://www.progeriaresearch.org). The following fibroblasts were used: HGADFN003, HGADFN127, HGADFN155, HGADFN164, and HGADFN188. Control fibroblasts were obtained from the Coriell Institute for Medical Research (Camden, NJ, USA). The following cell lines were used: GM01651C, GM0323B, GM03349C, GM03348E, and GM08398A. Cells were cultured as described previously (McClintock *et al*., 2006).

FTI lonafarnib (Merck USA) was added to the culture media at a concentration of 1.5 μm daily for 3–9 days, as reported previously (Marji *et al*., 2010). Sulforaphane (Sigma-Aldrich) was added to the media at a concentration of 1.0 μm. Mock-treated fibroblasts were cultured in parallel with media containing the vehicle (DMSO).

To block proteasome activity, 1.0 μm MG132 (Sigma-Aldrich) was applied for 12 h. To block lysosomal activity, 25 μm chloroquine (Sigma-Aldrich) was applied for 12 h in the presence or absence of 1 μm SFN.

### 2D-DIGE analysis of dermal fibroblast nuclear preparations

Two control (GMO3349C, GMO3348C) and two HGPS (HGADFN127, HGADFN003) cell lines in the growth phase were used. Each preparation of 10^7^ cells was resuspended in 5 mL of buffer A (10 mm HEPES/KOH pH 7.9, 1.5 mm MgCl_2_, 10 mm KCl, and 0.5 mm DTT) and broken with 12–15 strokes in a tight Dounce homogenizer. The nuclei were isolated by centrifugation at 800 ***g*** for 10 min at 4 °C. The pellets were resuspended in 10 mL of buffer B (10 mm Tris/HCl pH 7.5, 3.3 mm MgCl_2_ and 0.25 m sucrose) and centrifuged for 5 min at 1000 rpm. The resulting pellet was resuspended in 2.5 mL of 10 mm MgCl_2_ and 0.25 m sucrose, layered over a gradient containing 2.5 mL of 0.5 mm MgCl_2_ and 0.35 m sucrose, and centrifuged at 2500 rpm for 10 min. The purified nuclei were confirmed by microscopy. Nuclear pellets were separately resuspended in two-dimensional lysis buffer (30 mm Tris-HCl, pH 8.8, 7 m urea, 2 m thiourea, and 4% CHAPS) at concentrations between 4 and 6 mg mL^−1^. Control and HGPS samples were labeled separately with CyDy2 or Cy3 fluors. The labeled samples were mixed with 2 × 2-D sample buffer and loaded onto pH 3–10 linear IPG strips, isoelectric focusing (IEF) and further separated onto 12% SDS-polyacrylamide gels. Two independent experiments were performed.

Gel images were scanned using a Typhoon TRIO Imager (Amersham BioSciences), The scanned images were analyzed with imagequant software version 6.0 (Amersham BioSciences), followed by in-gel analysis using decyder software version 6.0 (Amersham BioSciences). The decyder spot detection algorithm ratio and threshold were set to a 1.5-fold change for calculations. We selected 40 protein spots in experiment 1 and 35 in experiment 2. Protein spots were collected with an Ettan Spot-Picker (Amersham BioSciences) using the decyder software. MALDI-TOF mass spectra were acquired, and TOF/TOF tandem MS fragmentation spectra were acquired for each sample. The resulting peptide masses were analyzed as described in supporting information. Candidates with either a protein score CI% or an ion CI% >95 were considered significant.

### Cell toxicity

Cell toxicity was determined using a Cell Tox Green kit (Promega, Mannheim, Germany) according to the manufacturer's instructions. A concentration of 1.0 μm SFN was selected for all experiments, as higher concentrations resulted in increased cell death.

### Cumulative population doubling determination

Cells were seeded in triplicate at a density of 1.5 × 10^5^ cells per 10-cm dish and cultivated in DMEM high glucose medium for 10 days. Cells were harvested, and the number of cells was measured with a CASY® Cell Counter (Roche, Penzberg, Germany). Cumulative population doublings (CPDs) were determined using the following formula: *n* = 3.32 (log cells harvested – log cells seeded) + X, where *n* = the final CPD number at the end of a given subculture, and X = the former CPD as described previously (Marji *et al*., 2010).

### Real-time PCR analysis

Total RNA was extracted from the cell pellets using an RNase Mini Kit (Qiagen, Valencia, CA, USA) as described previously (Marji *et al*., 2010). All cDNAs were synthesized from cellular RNA using Omniscript Reverse Transcriptase (Qiagen). RNA was isolated from dermal fibroblasts obtained from subjects with HGPS and unaffected individuals. Primers were designed using Primer 3 (http://frodo.wi.mit.edu/cgibin/primer3/primer3_www.cgi). The list of genes evaluated by RT–PCR, and their corresponding primers are shown in Table S1. Specificity of primers was verified by PCR.

Real-time PCRs were performed with Power SYBR Green PCR mastermix (Applied Biosystems), 300 nm of each primer, and 50 ng of template in a 20-μL reaction volume. Amplification was carried out using an Mx3000P Real-Time PCR Detection System (Stratagene) with an initial denaturation step at 95 °C for 10 min followed by 40 cycles of 95 °C for 35 s, 60 °C for 20 s, and 72 °C for 45 s. Three experiments were performed for each assay, and all samples were run in triplicate. GAPDH was used as an endogenous control, and relative quantification was performed by determining the real-time PCR signal of the experimental RNA samples in relation to the signal of the control. The 2(ΔΔ*C*_T_) method was used to calculate the relative changes in gene expression (Livak & Schmittgen, 2001).

### Western blot analysis

Cell pellets were resuspended in Laemmli sample buffer (BioRad), and Western blots were performed as described previously (McClintock *et al*., 2006). The membranes were incubated with primary antibodies: anti-lamin A/C [kindly provided by Dr. N. Chaudhary (1/5000) (Chaudhary & Courvalin, 1993), anti-progerin antibody (clone S9, 0.1 μg mL^−1^) (McClintock *et al*., 2007), anti-prelamin A antibodies (sc-6214, Santa Cruz Biotechnology, 1/1000), anti-proteasome S20 subunit C2 (ab22665, Abcam, 1/000), anti-Hsp27 (ab2790, Abcam, 1/2000), anti-ubiquitin (sc-8017, Santa Cruz Biotechnology, 1/3000), anti-LC3B (Sigma-Aldrich, 1/4000), anti-53BP1 (A300-272A, Bethyl, 1/1000)], anti-FHL-1 (sc-133580, Santa Cruz Biotechnology, 1/1000) anti-Rad51 (NBP2-32622, Novus Biological, 1/1000) anti-β-actin (Sigma-Aldrich, 1/5000) and anti-β-tubulin (Thermo Fisher, 1/2000). Then washed and incubated with a corresponding secondary antibody coupled to horseradish peroxidase (Jackson ImmunoResearch Laboratories). Proteins were visualized using a chemiluminescence detection system (ECL substrate; BioRad). Signals were analyzed with image lab software (BioRad). Protein signals were quantified by normalizing to β-actin as indicated.

### Immunocytochemistry

Fibroblasts were grown directly on coverslips. Cells were fixed in 100% methanol at −20 °C for 10 min and further processed for immunohistochemistry as described (McClintock *et al*., 2006). Primary antibodies used in this study were anti-progerin S9 (1 μg mL^−1^) (McClintock *et al*., 2007), anti-lamm A A/C (Chaudhary N, 1/500), anti-lamm A B1 (sc-6217, Santa Cruz Biotechnology, 1/50), anti-Lap2α (IQ175, Immunoquest Laboratories, 1/500), antinuclear pore complexes (Nup414, ab50008, Abcam, 1/600), anti-HP1γ (MAB3450, Millipore, 1/400), anti-γH2AX (JBW301, Millipore, 1/200), anti-53BP1 (A300-272A, Bethyl, 1/100), and anti-lamm A A (133A2, Abcam, 1/150). The secondary antibodies were affinity-purified Alexa Fluor 488 goat or donkey IgG antibodies (Molecular Probes) and Cy3-conjugated IgG antibodies (Jackson ImmunoResearch). All samples were also counterstained with DAPI in Vectashield mounting medium (Vector Inc.). Images were acquired using on an Axioplan fluorescence microscope (Carl Zeiss).

### Measurement of proteasome activity in fibroblasts

Treated and untreated cells were harvested and counted using CASY cell counting technology (Roche Innovatis AG). The cell numbers were adjusted to 2.6 × 10^5^ before processing, as suggested by instructions of the Cayman 20S Proteasome Assay Kit (Cayman Chemical Company). Briefly, equal numbers of cells from all samples were separately washed with 200 μL of assay buffer and incubated with 100 μL of lysis buffer for 30 min. Samples were then centrifuged for 10 min at 500 g, and 40 μL of supernatant from each sample was added to a 96-well plate. Additionally, 40 μL lysis buffer, 10 μL assay buffer, and 10 μL SUC-LLVY-AMC substrate were added to each well. The fluorescence intensity was measured at 360 and 480 nm to determine the 360/480 absorbance ratio.

### Autophagy measurements in fibroblasts

The autophagic vacuoles in treated and mock-treated fibroblasts were quantified using an Autophagy/Cytotoxicity Dual Staining Kit (Cayman Chemical Company). Cells were harvested, and equal numbers were seeded in triplicate in 96-well plates. The cells were allowed to attach at 37 °C for 12 h. The number of adherent cells after 12 h was determined prior to assays in parallel wells. Monodansylcadaverine (MDC) was added to the wells at a 1:1000 ratio for a final volume of 100 μL. Measurements of the autophagic vacuole intensities were obtained using an excitation wavelength of 335 nm and an emission wavelength of 512 nm.

### Measurement of ROS in fibroblasts

Reactive oxygen species levels were measured using 2′,7′-dichlorofluorescein diacetate (DCFDA) with the Cellular ROS Detection Assay Kit from Abcam according to the manufacturer's instructions. Briefly, mock-treated and SFN-treated cells were seeded in a 96-well plate for 12 h. The number of adherent cells was determined prior to assays. Cells were then incubated with 25 μm DCFDA for 45 min at 37 °C and washed, and the fluorescence was measured using a VICTOR3 1420 Multilabel Counter (PerkinElmer).

### Measurements of ATP in fibroblasts

The intracellular ATP content of treated and mock-treated fibroblasts was measured using a CellTiter-Glo Luminescent Cell Viability Assay (Promega). Cells were harvested and seeded at equal densities in triplicate in 96-well plates. These cells were allowed to attach at 37 °C for 12 h and were counted prior to assays. Cells were then incubated with 100 μL of CellTiter-Glo reagent (CellTiter-Glo buffer plus CellTiter-Glo substrate) for 10 min and the luminescence intensity was measured. An ATP standard was assessed in parallel.

### Statistical analyses

For all the experiments, the results are presented as the mean ± SD. Comparisons were performed using Student's *t*-test. A *P* value of *P* < 0.05 was considered statistically significant. Sample sizes are indicated in the figure legends.

## Supporting Information

**Fig. S1**Cytotoxicity of sulforaphane. **Fig. S2** Sulforaphane restores the levels of FHL-1 in HGPS cells. **Fig. S3** Sulforaphane restores the levels of Rad 51 in HGPS cells. **Fig. S4** The combination of SFN and an FTI does not exert a synergistic effect on HGPS cell FTI. **Table S1.** List of primers used for real-time PCR. **Appendix S1.** Supporting Information to Experimental Procedures.
