# Supplementary material for: Sulforaphane enhances progerin clearance in Hutchinson–Gilford progeria fibroblasts
Source: Aging Cell. 2014 Dec 16;14(1):78–91. doi: 10.1111/acel.12300 (PMC4326906; doi:10.1111/acel.12300)
Supplement: Supplementary file 1 [file acel0014-0078-sd1.docx]

# Supporting Information

Sulforaphane enhances progerin clearance in Hutchinson-Gilford progeria fibroblasts

Diana Gabriel^1^, Daniela Roedl^1^, Leslie B. Gordon^2^ & Karima Djabali^1*^

^1^ Department of Dermatology and Institute for Medical Engineering, Technische Universität München (TUM)

^2^Department of Pediatrics, Alpert Medical School of Brown University and Hasbro Children’s Hospital, Providence, RI, USA; Boston Children’s Hospital and Harvard University, Boston, MA

^*^Corresponding author

Karima Djabali

Technische Universität München

Tel 49 89 289 10920

Fax 49 89 289 10928

email:djabali@tum.de

# Supporting Information

**Table S1:** List of primers used for real-time PCR and qPCR

| Primer | Primer sequence |
| --- | --- |
| STUB1 F  STUB1 R | 5’-CTGCTGTTGGACTGTGGACT-3’  5’-CTCGTGCTCACGGATTTTAT-3’ |
| BAG3 F  BAG3 R | 5’-GGAGTGCTGAAAGTGGAAGC-3’  5’-CTGGACTTGACCTGGGACAT-3’ |
| BAG2 F  BAG2 R | 5’-GCTTTGAGAGAAGCAGCAAC-3’  5’-GCTGGGGGTTTCTAATTGTT-3’ |
| BAG1 F  BAG1 R | 5’-AGCAATGAGAAGCACGACCT-3’  5’-GAAGTGCACCATGGAGAGGAG-3’ |
| Hsp27 F  Hsp27 R | 5’-GTCCCTGGATGTCAACCACT-3’  5’-gacagggaggaggaaacttg-3’ |
| Hsp70 F  Hsp70 R | 5’-GCTCTTTGCTGCTTCACTTC-3’  5’-AGGTGGCAGTGTTGATTCAT-3’ |
| Hsp90a F  Hsp90a R | 5’-GTCTAGTTGACCGTTCCGCA-3’  5’-GAGGAGGCACCCTCAAGTTC-3’ |
| Hsp90b F  Hsp90b R | 5’-GAAGTGCACCATGGAGAGGAG-3’  5’-GCGAATCTTGTCCAAGGCATC-3’ |
| LMNA/C F  LMNA/C R | 5’-GCAAAGTGCGTGAGGAGTTT-3’  5’-GAGTTCAGCAGAGCCTCCAG-3’ |
| Progerin F  Progerin R | 5’-ACTGCAGCAGCTCGGGG-3’  5’-tctgggggctctgggc-3’ |
| LMNA F  LMNA R | 5’-GTGAGTACAACCTGCGCTCG-3’  5’-GAGTGACCGTGACACTGGAG-3’ |
| GAPDH F  GAPDH R | 5’-CTCTGCTCCTCCTGTTCGAC-3’  5’-TTAAAAGCAGCCCTGGTGAC-3’ |
| Lamin B1 F  Lamin B1 R | 5’-AAGCAGCTGGAGTGGTTGTT-3’  5’-TTGGATGCTCTTGGGGTTC-3’ |


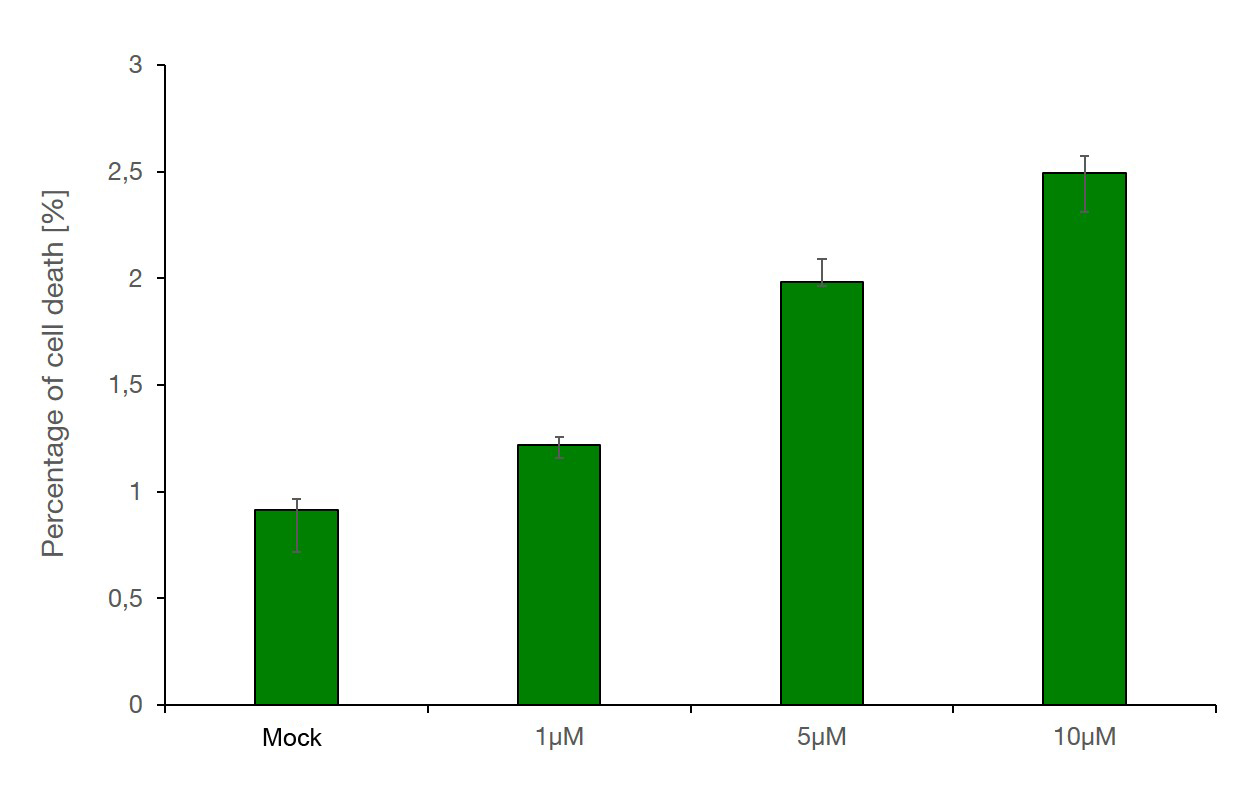


**Fig. S1:** **Cytotoxicity of Sulforaphane**. Control cells were incubated for 48h with increasing concentrations of sulforaphane (0 to 10 µM, as indicated, stock solution was made in DMSO) diluted in complete culture medium. To mock treated cells, the vehicle alone was added. The percentage of dead cells was determined using a Cell Tox Green kit as described in the Procedures. All values are presented as mean ± S.D; n=3.


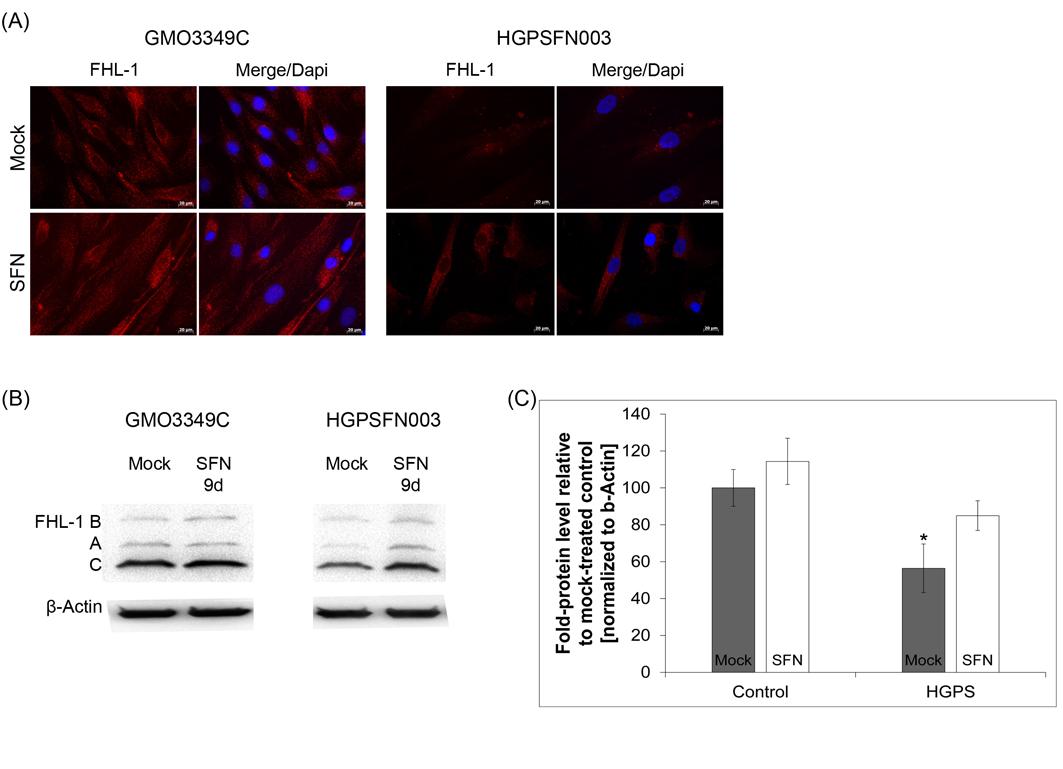


**Fig. S2:** **Sulforaphane restores the levels of FHL-1 in HGPS cells.**

(A) Immunocytochemistry using an anti-FHL-1 antibody was performed on normal (GMO3349C) and HGPS (HGADFN003) cells mock-treated or SFN-treated cells for 9 days. Scale bar: 20 μm. (B) Western blot evaluation of FHL-1 levels in control and HGPS cells that were treated as in (A). (C) Quantification of FHL-1 levels normalized to β-actin and presented as the fold change relative to control cells (*p<0.05; n=3).

**
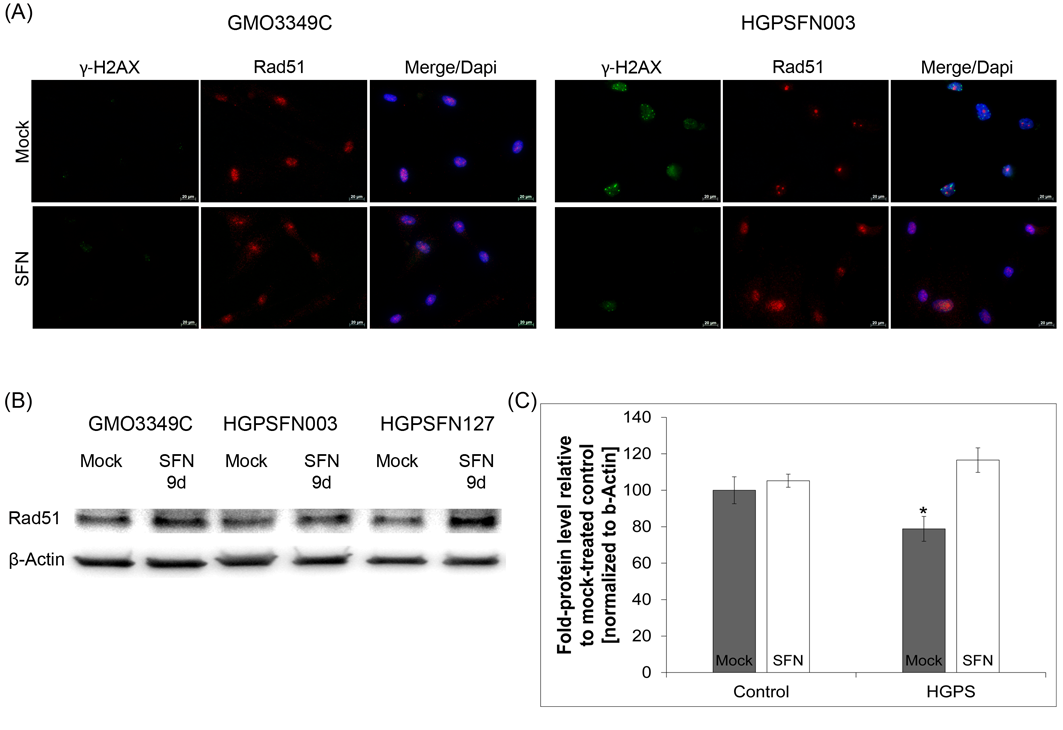
**

**Fig. S3: SFN restores the levels of Rad51 in HGPS cells.**

(A) Immunocytochemistry using antibodies directed against the indicated proteins (γ-H2AX and Rad51) was performed on normal (GMO3349C) and HGPS (HGADFN003) cells mock-treated or SFN-treated cells for 9 days. Scale bar: 20 μm. (B) Western blot evaluation of Rad51 levels in control and HGPS cells that were treated as in (A). (C) Quantification of Rad51 levels normalized to β-actin and presented as the fold change relative to control cells (*p<0.05; n=3).

**
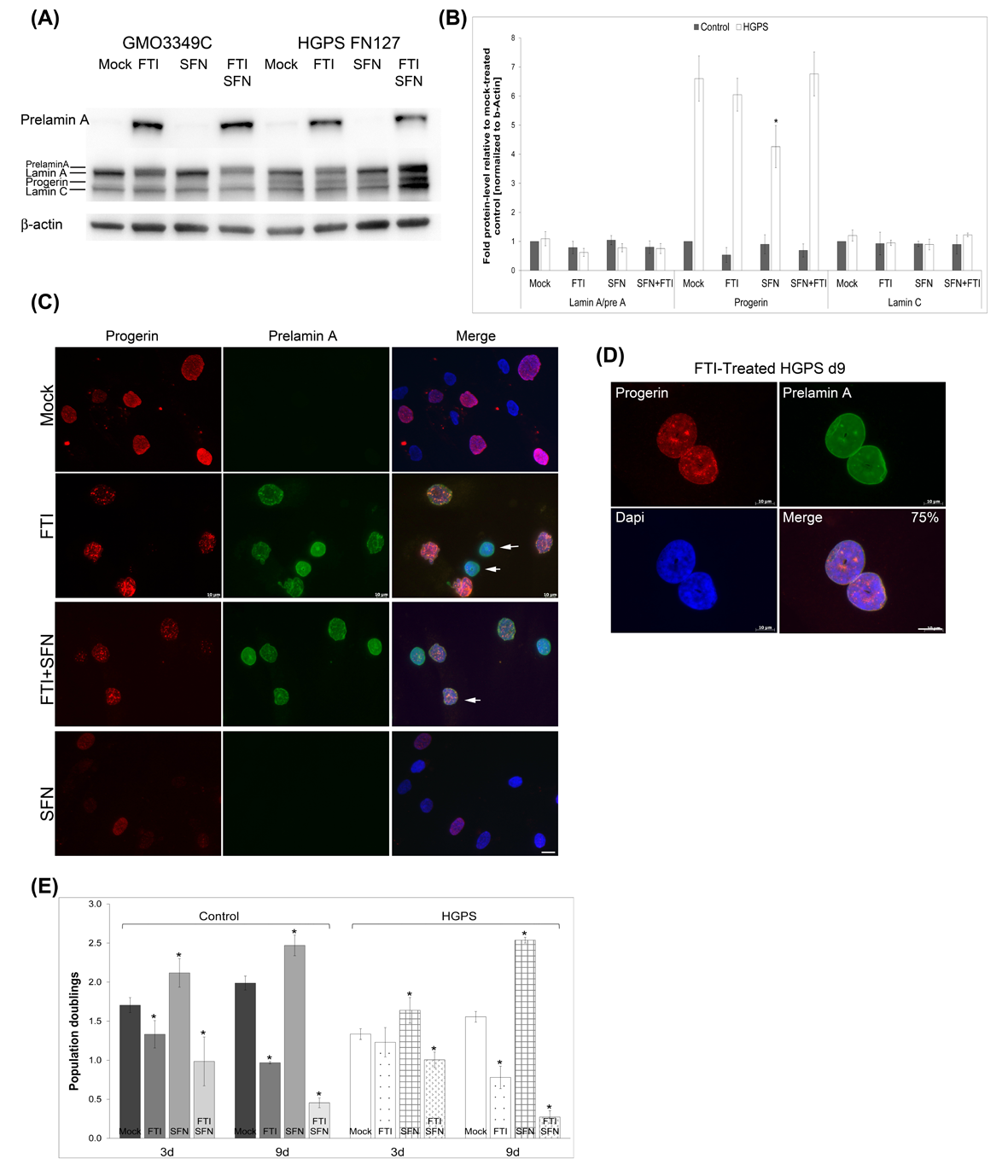
**

**Fig. S4: The combination of SFN and an FTI does not exert a synergistic effect on HGPS cells.**

(A) Western blot evaluation of A-type lamins (prelamin A, lamin A, lamin C and progerin) levels in control and HGPS cells that were mock-treated, FTI-treated, SFN-treated or treated with a combination of SFN plus FTI daily for a period of 4 days (a representative image is shown; n=4). Blots were probed with antibodies against prelamin A, lamin A/C and β-actin. (B) Densitometric analysis of lamin A/prelamin A, lamin C and progerin signals. Data represent the mean ± S.D. with respect to mock-treated control cells after the values were normalized to the β-actin signal (n=4). (C) Localization of progerin and prelamin A by indirect immunofluorescence in HGPS cells. HGPS cells were mock treated, or treated with FTI (1.5 μM), SFN (1μM) or a combination of FTI (1.5 μM) plus SFN (1μM) daily for a period of 4 days. The merged images correspond to progerin, prelamin A and Dapi signals. The arrows indicate donut-shaped nuclei. Scale bar: 10 μm. (D) High-magnification images of donut-shaped nuclei in HGPS cells treated with FTI (1.5 μM) for 9 days. The cells were labeled as indicated. Analysis indicated that 75% of the donut-shaped nuclei were binucleated in HGPS cells after 9 days of FTI treatment. Scale bar: 10 μm. (E) Population doublings were calculated as stated in the Materials and Methods for control and HGPS cells that were mock treated (DMSO) or treated daily with 1.5 μM FTI, 1 μM SFN, or both for a period of 3 or 9 days.

### Supporting Information to Experimental Procedures

### Two-dimentional difference in gel electrophoresis (2D-DIGE) analysis of dermal fibroblast nuclear preparations.

We selected 40 protein spots in experiment 1 and 35 in experiment 2. Protein spots were collected with an Ettan Spot-Picker (Amersham BioSciences) based on the in-gel analysis and spot picking design generated by the DeCyder software. The gel spots were washed and digested in-gel with modified porcine trypsin protease (Trypsin Gold, Promega). The digested tryptic peptides were desalted using a Zip-tip C18 (Millipore). The peptides were eluted from the Zip-tip with 0.5 µl of matrix solution (Agilent Technologies) and spotted on the matrix-assisted laser desorption/ionization (MALDI) plate (model ABI 01-192-6-AB). MALDI-time of flight (TOF; MS) and TOF/TOF (tandem MS/MS) were performed on an ABI 4700 mass spectrometer (Applied Biosystems). MALDI-TOF mass spectra were acquired in the reflection-positive ion mode, averaging 4,300 laser shots per spectrum. TOF/TOF tandem MS fragmentation spectra were acquired for each sample, averaging 4,300 laser shots per fragmentation spectrum for each of the ten most abundant ions in each sample (excluding trypsin autolytic peptides and other known background ions). The resulting peptide masses and the associated fragmentation spectra were entered into GPS Explorer software version 3.5 equipped with a MASCOT search engine (Matrix Science) to search the National Center for Biotechnology Information non-redundant (NCBInr) database. The parameters were set at 800-4,000 Da to create the “peak list.” Searches were performed without constraining the protein molecular weight or isoelectric point, with variable carbamidomethylation of cysteine and oxidation of methionine residues, and with 1 missed cleavage permitted in the search parameters. The mass tolerance was set at 0.3 Da and 100 ppm. Candidates with either a protein score confidence interval (CI)% or an ion CI% greater than 95 were considered significant.
